# The Use of Genetically Engineered Mouse Models for Studying the Function of Mutated Driver Genes in Pancreatic Cancer

**DOI:** 10.3390/jcm8091369

**Published:** 2019-09-02

**Authors:** Ching-Chieh Weng, Yu-Chun Lin, Kuang-Hung Cheng

**Affiliations:** 1 Institute of Biomedical Sciences, National Sun Yat-Sen University, Kaohsiung 804, Taiwan; 2 Department of Medical Laboratory Science and Biotechnology, Kaohsiung Medical University, Kaohsiung 807, Taiwan

**Keywords:** Pancreatic cancer, Genetically engineered mouse models (GEMMs)

## Abstract

Pancreatic cancer is often treatment-resistant, with the emerging standard of care, gemcitabine, affording only a few months of incrementally-deteriorating survival. Reflecting on the history of failed clinical trials, genetically engineered mouse models (GEMMs) in oncology research provides the inspiration to discover new treatments for pancreatic cancer that come from better knowledge of pathogenesis mechanisms, not only of the derangements in and consequently acquired capabilities of the cancer cells, but also in the aberrant microenvironment that becomes established to support, sustain, and enhance neoplastic progression. On the other hand, the existing mutational profile of pancreatic cancer guides our understanding of the disease, but leaves many important questions of pancreatic cancer biology unanswered. Over the past decade, a series of transgenic and gene knockout mouse modes have been produced that develop pancreatic cancers with features reflective of metastatic pancreatic ductal adenocarcinoma (PDAC) in humans. Animal models of PDAC are likely to be essential to understanding the genetics and biology of the disease and may provide the foundation for advances in early diagnosis and treatment.

## 1. Introduction

Pancreatic ductal adenocarcinoma (PDAC) is the most deadly of common human adult malignancies [1]. The vast majority of patients present with unresectable disease, and have virtually no hope for cure or even long-term survival [2,3]. This advanced clinical presentation has also resulted in extremely limited tissue resources for biological investigations of these tumors. Despite significant advances in the past two decades in the chemotherapeutic management of human malignancies, there has been only very slight impact on the extremely poor median survival of patients with PDAC [1,4,5].

**Figure 1:**
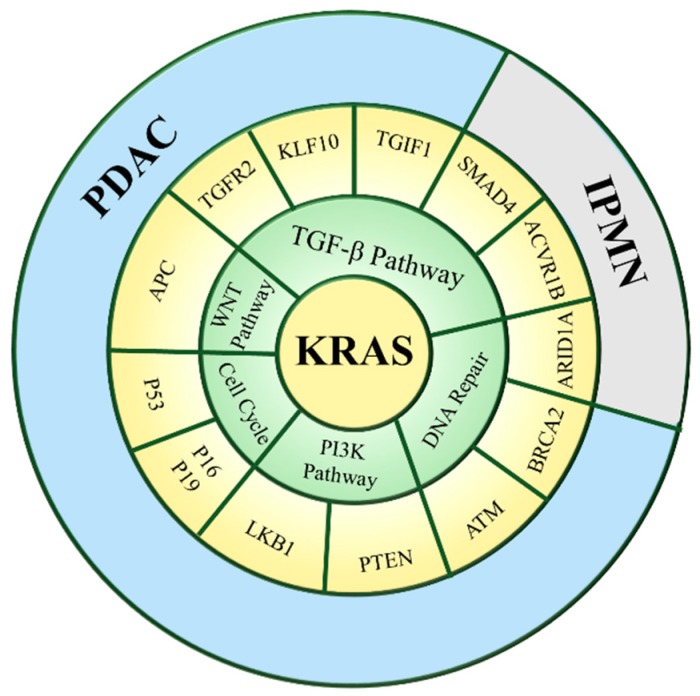
Cross talk of mutant Kras signaling with other altered cellular pathways in pancreatic ductal adenocarcinoma (PDAC). Five core cellular pathways and processes have been identified that are altered through gene mutations with high frequency in PDAC. Thirteen representative mutated genes associated with each pathway are depicted in the outermost circle. The most common mutation of KRAS signaling pathway is located in the center. IPMN: Intraductal papillary mucinous neoplasm.

**Figure 2:**
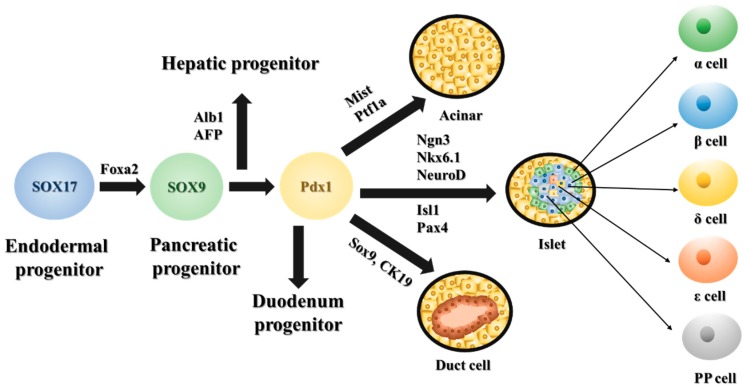
Schematic diagram of key transcription factors regulating pancreas development. During early pancreas specification and lineage commitment, specific transcriptional factors and other critical markers are expressed at indicated stages. Pancreatic progenitors give arise to the three major pancreatic lineages—ducts, acini, and islets.

Amidst these dismal statistics, there are three areas of recent significant progress in understanding pancreatic carcinogenesis. The first is the observation that PDACs arise from the progression of non-invasive ductal epithelial neoplasms. Termed pancreatic intraepithelial neoplasms (PanIN), these lesions have progressively increasing architectural and cytological atypia, akin to intraepithelial neoplasms in other human tissues [6,7]. Graded on a scale of 1 to 3, the presence of these neoplasms suggests a target lesion for screening, early diagnosis and possibly chemoprevention. The second area of discovery is in the delineation of several key genetic alterations (signature lesions) that typify the development of most human PDACs. Pancreatic adenocarcinomas display a characteristic profile of genetic lesions, consisting of mutations in INK4A, KRAS, SMAD4/DPC4, and TP53 in a high proportion of tumors, and less frequent mutations in LKB1, APC, CTNNB1, ATM, BRCA2, ACVR1B, MKK4 (Ras downstream effector), and ARID1A [8,9]. Ongoing studies have been directed at determining the biological roles of these PDAC driver gene mutations, and in particular, relating those alterations to the processes of cancer initiation and progression (Figure 1). Consistent with the model that PDACs arise from PanIN progression, those signature lesions have also been identified in non-invasive precursors PanINs [10]. The identification of many additional oncogenic alterations has more recently been elucidated with transcriptional and genomic profiling technologies, suggesting that more significant advances in biological understanding are forthcoming. The third important advance is the development of genetic manipulation tools to engineer mice with PDAC. Pancreatic progenitor cells are characterized by the expression of many transcriptional factors, such as Sox17, Foxa2, Sox9, Pdx1, Ptf1a (p48), Pax4, Nkx6.1, and Ins1, which can differentiate into three distinct cell types of the pancreas, including exocrine, endocrine, and ductal cells [11,12]. Subsequently, the pancreatic endocrine lineage is triggered by the transient activation of neurogenin3 (Ngn3) transcriptional regulator transiently expressed at E12.5, and results in the generation of different hormone-expressing cell types (β-cells, α-cells, δ-cells, and PP cells) (Figure 2). One of the most common ways to target pancreatic progenitor cells and to achieve selective genetical modifications is the Pdx1-Cre transgene, which was developed in the Melton lab. It directs Cre recombinase to the pancreatic lineages around embryonic day 8.5, to both activate or abrogate gene function in a pancreas-specific manor [12,13]. In addition to Pdx-1 Cre strain, other groups also utilize P48 (Ptf1a), Sox9, Ngn, Pax4, or Ins1 Cre transgenes to design conditional pancreas specific mouse models (Figure 2). The engineering of Cre recombinase under a pancreas-specific promoter (Pdx1-Cre) has been utilized to generate mice with Kras activation and Ink4a/Arf or P53 inactivation, simulating key lesions in human PDAC [7,14,15]. These mice develop not only PDACs, but also progressive non-invasive atypical epithelial lesions analogous to human PanINs [7,16]. These genetic lesions engage common oncogenic signaling pathways in the pathogenesis of the human disease, hence the mouse model should provide a relevant system for elucidating the molecular circuitry of human pancreatic adenocarcinoma. Here, we summarize latest reports describing different PDAC models and present detail insight into such genotype-phenotype correlations—and of the associated molecular circuitry driving these processes—critical for both the design and assessment of efficacy of targeted therapies. 

## 2. Modeling K-RAS Mutation in the Pancreas

KRAS is an oncogene that encodes a small GTPase transductor protein that, in its active, GTP-bound form, engages a broad series of kinase pathways relating to cell proliferation, survival, migration, metabolism, and many other biological processes. Numerous pathways, such as stimulated growth factor receptors, transduce their downstream effects through RAS guanine exchange factors (RAS-GEFs) that activate the RAS family proteins, KRAS, HRAS, and NRAS [17,18]. Conversely, negative regulatory pathways induce RAS GTPase activator proteins (RAS-GAPs) that attenuate RAS signaling. Oncogenic KRAS mutations—at codons 12, 13 and 61—turn constitutively active KRAS forms, obviating the need for upstream inducing signals and rendering the protein insensitive to inhibition [19,20]. Activated KRAS engages multiple effector pathways, notably the RAF-mitogen activated kinase (MAP-kinase), phosphoinositide-3-Kinase and RalGDS pathways [21,22].

In addition to a role in tumor initiation, it appears that KRAS activation is required for maintenance of the tumorigenic growth of established PDAC, since disruption of KRAS activity—via RNA interference, antisense RNA, or expression of dominant-negative KRASN17—attenuates the tumorigenicity of PDAC cell lines [23]. Hence, KRAS activity seems to be required during all phases of pancreatic ductal tumorigenesis, and thus activated KRAS, or its effectors, are likely to be appropriate targets for the prevention and treatment of this malignancy [24]. It is notable, however, that the biochemical pathways induced by KRAS, and the resulting impact on cellular phenotypes, probably vary at different stages of tumorigenesis depending on the presence of other oncogenic mutations, on changes in intersecting signaling pathways and on other alterations in the cellular context. For example, phenotypes, such as enhanced proliferation and invasive growth—known to be associated with RAS activity—are restricted to the later stages of pancreatic neoplasia. Important future work is needed to resolve the context-dependent activities of KRAS and its signaling surrogates [24]. The elucidation of the critical KRAS effectors mediating PDAC pathogenesis and the specific biological processes provoked by KRAS signaling remain highly significant challenges in understanding the progression of this disease and in enabling the selection of effective drug targets [25,26,27].

Activating K-Ras mutations are the first genetic changes detected in the progression series present in about 30% of lesions showing the earliest stages of histological disturbance [28]. Increasing in frequency with disease progression, K-Ras mutations are found in nearly 90% of PDACs, and thus this lesion appears to be a required event for this malignancy [29]. The mouse model has validated the role of K-Ras activation in the initiation of PanIN and the insufficiency of this mutation in inducing malignant progression [30]. The role of KrasG12D on the initiation of pancreatic neoplasia was confirmed through analysis of clinically healthy Pdx1-Cre; LSL-Kras^G12D^ mice from eight to 26 weeks of age, all showing pancreatic ductal lesions strongly reminiscent of human PanINs. Pdx1-Cre-mediated activation of the KrasG12D allele alone leads to PanIN formation, the constellation of mucinous transformation of the ductular epithelium with nuclear atypia, and papillary growth [7,24]. Again, although Pdx1-Cre mediates recombination in all pancreatic lineages, the phenotype of Pdx1-Cre; LSL-Kras^G12D^ mice is relatively restricted to these abnormal ductal structures. Similar results were observed when crossing KRAS^G12D^ mice with animals harboring Cre recombinase under the control of pancreatic acinar cell-specific promoter, p48 (p48Cre), yields KRAS^G12D^; p48Cre mice that develop pancreatic intraepithelial precursor lesions (PanINs) within four weeks of age [13,24]. Thus, these data might imply that the acinar cells play an important role in response to oncogenic mutant KRAS mediated transformation in pancreas. Over time, a few of these PanINs may eventually progress to PDAC. Thus, Kras activation in the correct cellular compartment should recapitulate similar findings. Finally, this model allows cellular changes to be observed during the progression of the tumor phenotype, during a convenient experimental window of under three months [7]. Thus, the cell population that is demonstrated to support PanIN formation following Kras activation can be tested for its ability to support PDAC development following the superimposed deletion of Ink4/Arf, p53, SMAD4, or other mutations within that compartment [7,15,31].

## 3. PI3K/AKT Activation in PDAC

PI3K–AKT Pathway is an intracellular molecular pathway that plays a role in the regulation of cell proliferation, survival, and metabolism [32,33]. It can be activated by a multi-step process involving phosphatidylinositide (PtdIns) phosphate-mediated recruitment of AKT and its upstream kinases, including 3-Phosphoinositide-dependent kinase 1 (PDK1), to the inner surface of the cell membrane [34]. In vitro and xenograft studies have provided evidence that PI3K is critical for PDAC pathogenesis. Meanwhile, AKT is constitutively active in primary PDACs, and in xenografts and disruption of the PI3K–AKT pathway in cell lines. Along with chemical inhibitors or expression of dominant-negative AKT mutants, it interferes with cell growth, survival, and response to chemotherapy [35,36,37,38]. The mutant activated KRAS, the defining mutation hot spot in PDAC, may activate the PI3K/AKT pathway directly, or through promoting autocrine EGFR signaling. Signaling through other growth factor receptors, such as the Insulin-like growth factor-1 receptor (IGF-1R), may also contribute to the activation of PI3K/AKT signaling [35,39]. Notably, mutations in PTEN do not appear to contribute to AKT activation in PDAC, although some tumors may have reduced PTEN expression levels [37,40]. In addition, amplification of regions of chromosome 19 spanning the AKT locus are detected in some PDAC cell lines and primary tumors, and correlate with high relative levels of AKT expression, suggesting that gene copy increases may contribute to elevated AKT activity in some PDACs [41]. In addition, another tumor suppressor kinase, LKB1, also named STK11, is also known to interact with phosphorylated PTEN to increase its stabilization, and influence the phosphoinositide 3-kinase (PI3K)/Akt pathway [42,43,44]. Meanwhile, Romain Baer and his colleagues showed that hitting PI3K p110α subunit activity using a kinase-dead model and not hitting its expression is sufficient to completely prevent the initiation of pancreatic ductal cancers in a dose-dependent manner [45]. Their results supported that p110α is also a target in p53 mutant PDAC. In contrast to EGFR deletion, deletion of p110α completely protects from oncogenic Kras and mutated p53-induced PDAC and lethality [46,47].

### 3.1. Ptf1aCre, KrasG12D, EgfrKO Mice

PDACs show elevated expression of EGF receptors and their ligands, consistent with the presence of autocrine EGFR signaling [48,49]. EGFR and HER2/neu signaling pathways, induced in low grade PanINs, are among the first markers of pancreatic cancer progression, suggesting that autocrine EGF family signaling may contribute to the earliest stages of pancreatic ductal neoplasia [50,51]. The EGFR pathway is also likely to contribute to the maintenance of established tumors, since disruption of EGFR signaling in human PDAC cells inhibits growth in vitro and tumorigenesis in xenografts. The oncogenic effects of EGFR signaling in PDAC are likely to be directed by numerous effectors (including PI3-kinase and NFκB) that regulate tumor angiogenesis, as well as cell autonomous survival and proliferative processes [52,53]. Animals carrying floxed alleles of the EGFR locus did not develop PanIN lesions or PDAC tumors even in the context of pancreatic injury (pancreatitis) or lacking the p16Ink4a/p19Arf tumor suppressors [46,47]. However, mouse models from others further indicated that aberrant activation of EGFR signaling in the pancreas produces metaplastic change of the pancreatic ducts, lesions that subsequently evolve into benign cystadenomas in the context of p53 and/or Ink4a/Arf loss [54,55]. However, neither PanIN or PDAC are generally observed indicating that EGFR activation may play a surrogate role in PDAC pathogenesis—perhaps in concert with activated KRAS—rather than operating in a dominant oncogenic fashion. 

### 3.2. Pdx1-Cre, KrasG12D/+, Pten^L/+^ Mice

Aberrant activation of the PI3K–AKT pathway has been widely implicated in human cancers, including PDAC [56]. The PTEN tumor suppressor gene encodes a phosphatase that dephosphorylates the PI3K product PIP3, and thereby terminates PI3K signaling; that provided the first strong evidence that PI3K signaling may be widely implicated in human cancer [57,58,59]. While mutation of PTEN is not common in pancreatic cancer, a decrease or loss of PTEN expression has been reported in up to 60% of pancreatic cancer cell lines or tumor tissues [60]. In vitro studies demonstrated that haploinsufficiency of Pten, a negative regulator of the PI3 kinase pathway, was sufficient to allow constitutive activation of Akt in many different cancer cells [61]. Consequently, the potential role for PTEN as a haplo-insufficient tumor suppressor is further supported by several mouse genetic studies [62]. A previous work with mice with only Pten deficiency in the pancreas revealed that loss of PTEN function in Pdx1-Cre, Pten^Lox/Lox^ mice led to a centroacinar cell that possessed adult stem cell properties, the expansion of which produced metaplasias similar to the phenotypes of Pdx1-Cre, Kras^G12D/+^, Pten^Lox/+^ mice. In addition to the intensive metaplastic phenotype, Pdx1-Cre Pten^lox/lox^ mice eventually develop invasive pancreatic adenocarcinomas at a low frequency [63]. Meanwhile, Pdx1-Cre, Pten^Lox/Lox^, p53^−/−^ compound mice tend to develop papillary ductal adenocarcinomas (age, 4–6 months), sometimes mixed with small acinar carcinoma features [64].

### 3.3. Pdx1-Cre, LKB1^L/L^ Mice

LKB1 (Liver kinase B1) is a tumor suppressor gene that activates the AMPKα signaling pathway and regulates cell polarity, survival, and metabolism [43,65]. Inactivation or down regulation of LKB1 gene has been observed in a numerous of human cancers, including pancreatic cancer. In addition, germline mutations in LKB1 have been associated with Peutz–Jeghers syndrome, which comprises gastrointestinal polyps and increases more than 100-fold, the risk of developing pancreatic cancer. Knockdown of LKB1 has been reported to increase cell growth, migration, invasion, and chemoresistance in PDAC. Moreover, as conventional knockout of Lkb1 in mice leads to embryonic lethality, conditional deletion of the LKB1 gene in the pancreas was generated and demonstrated the presence of cystic neoplasms that resemble human mucinous cystic neoplasms (MCNs) [66,67]. Moreover, LKB1 haploinsufficiency cooperating with K-rasG12D mutation in mice leads to increased incidences of PanINs and PDAC in compared to K-rasG12D mutation ones [66]. That evidence indicated the critical tumor suppressor role of LKB in pancreatic cancer progression.

## 4. Modeling Cell Cycle Inhibitors INK4a/ARF Loss in PDAC

Loss of INK4A function, brought about by mutation, deletion, or promoter hypermethylation, occurs in 85%–90% of sporadic PDACs [29]. INK4A loss is generally seen in moderately advanced lesions that show features of dysplasia. The dissection of the role of INK4A has been a fascinating story, since this gene is a resident of the INK4A/ARF tumor suppressor locus at 9q21, a locus which also encodes the ARF tumor suppressor via distinct first exons and alterative reading frames in shared downstream exons [68,69]. Given this physical juxtaposition and frequent homozygous deletion of 9p21 (approximately 40% of tumors), many PDACs sustain loss of both INK4A and ARF, thereby disrupting both the RB and p53 tumor suppression pathways. In humans, INK4A appears to be the more important pancreatic cancer suppressor of this locus, as evidenced by germline and sporadic mutations that target INK4A but spare ARF [70,71]. INK4A loss is observed in some early PanIN (~20% of PanIN-1A and ~30% of PanIN-1B) and at increasing levels in higher grade PanINs. Nabeel and his colleagues investigated the role of the Ink4a/Arf locus in suppression of pancreatic ductal neoplasia in the mice and have found that Ink4a/Arf deletion alone does not give significant predisposition to PDAC but that, in the context of Kras activation, Ink4a/Arf deficiency leads to the rapid progression of PanIN to metastatic PDAC. In numerous in vitro and in vivo systems, RAS alleles have been shown genetically and biochemically interact with both INK4A and ARF [72,73,74]. Specifically, Ink4a and/or Arf deficiency effectively cooperates with mutant Kras^G12D^ alleles in promoting tumors in several mouse models [75,76]. In the context of PDAC pathogenesis, it should be important to determine what signals, if any, provoke INK4A and ARF expression in PanIN of various stages, and to identify specific roles of either gene in restraining PanIN progression. In this model, LSL-KrasG^12D^, Pdx1-Cre, Ink4a/Arf^Loxp/Loxp^ mice with documented efficient deletion of the Ink4a/Arf in the pancreas developed weight loss, ascites, jaundice, and a palpable abdominal mass between seven and 11 weeks of age [7]. Autopsies revealed the presence of solid pancreatic tumors, ranging in diameter from 4 to 20 mm; in some cases, more than one distinct tumor nodule was apparent, suggesting multifocal disease. The tumors were highly invasive, frequently involving the duodenum and/or spleen and occasionally obstructing the common bile duct; however, gross and microscopic metastases to liver and lung were not evident [7]. This phenotypic comparison of the Pdx1-Cre, LSL-Kras^G12D^ mice with the Pdx1-Cre, LSL-Kras^G12D^, Ink4a/Arf^Loxp/Loxp^ mice unequivocally proves a role of Ink4a/Arf in constraining the malignant progression of early-stage ductal neoplasms. 

## 5. Aberrant TGFβ pathway in PDAC

**Figure 3:**
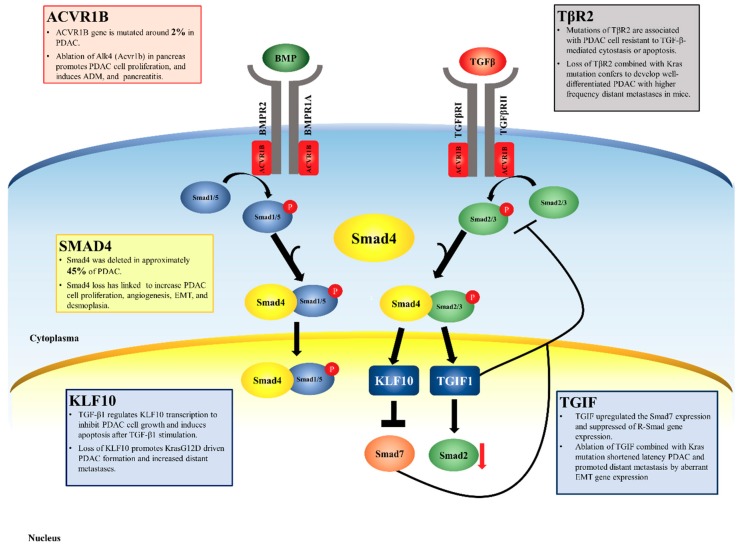
The impacts of mutations in the TGFβ signaling transduction components contribute to PDAC development. Overview of the dysregulation TGF-β/BMP signaling pathways in PDAC. In canonical TGFβ/Smad pathway, the receptor complexes of TGFβRI and TGFβRII are phosphorylated upon TGF-β binding and then activate Smad2/3 (R-Smad). Those activated R-Smads form a complex with the Co-Smad Smad4 and this complex is imported into the nucleus where in association with other transcription factors to regulate transcription of TGFβtarget genes. The TGFβ/BMP pathways’ components that are mutated, deleted, or downregulated, are listed and described in the boxes.

TGFβ is a potent inhibitor of epithelial cell growth and survival, although these effects are highly dependent on the cell context. In numerous epithelial cell lines and in epithelial tissue in vivo, TGFβ exerts a growth inhibitory program that involves modulation of cell cycle regulators, including the induction of p15INK4B and p21CIP1 expression, repression of c-Myc and ID family transcription factors, induction of apoptotic machinery, and repression of telomerase. The tumor suppressor role of TGFβ signaling is underscored by presence of inactivating TGFβ receptor mutations in a number of cancers [77]. Significantly the role of TGFβ in blocking cancer development also involves control of epithelial cell/tissue microenvironment interactions, as demonstrated by the development of epithelial cancers in mice with T-cell specific Smad4 deletion and fibroblast-specific TGFβRII deletion [78,79]. Finally, it should be noted that inactivation of BMP and Activin signaling are also implicated in cancer development, hence the tumor suppressor function of Smad4 may also involve a requirement in mediating signaling from these receptors (Figure 3). On the other hand, TGFβ can enhance the malignant growth of some established epithelial tumors, promoting tumor cell proliferation, migration, and the epithelial-to-mesenchymal transition (EMT)—a process by which advanced carcinomas acquire a highly invasive and undifferentiated phenotype and become metastatic [80,81]. Therefore, TGFβ signaling can have biphasic stage-specific effects—inhibiting carcinoma-initiation, while promoting the high-grade advancement and dissemination of established tumors. The significant crosstalk of Smad signaling with other mitogenic and survival pathways is likely to contribute to the switch from a cytostatic TGFβ program to a pro-tumorigenic program. Possible mechanisms include inactivation of the Rb pathway, altered protein–protein interactions with Smad binding proteins (such as Foxo proteins and Myc) and altered Smad protein stability. Possibly, differences in receptor trafficking may also contribute to regulation of PDAC tumorigenesis [82]. 

### 5.1. Pdx1-Cre, KrasG12D/+, SMAD4^L/L^ Mice

The 4th most frequently mutated gene in PDAC is SMAD4 (previously designated Delete in Pancreatic Cancer 4; DPC4), encoding a transcriptional regulator that is a central component in the TGF-β superfamily signaling cascades [83,84,85]. This gene maps to human chromosome 18q21, a region that sustains deletion in approximately 30% of PDAC cases. Inactivating mutations in Smad4 are far more common in PDAC than in any other cancer type [86]. The biological role of Smad4 mutations in human PDAC progression is an area of active investigation, often with contrasting observation. SMAD4 appears to be involved in progression since its loss occurs only in late-stage PanINs. As a central component of TGF-β signaling, SMAD4 status is likely to exert a prominent effect on host-tumor cross-talk relevant to cancer progression (Figure 3). The roles of TGF-β/Smad signaling in PDAC pathogenesis are not well defined and have been linked to cancer biological processes, such as cell proliferation, angiogenesis, epithelial-mesenchymal transitions, and a robust stromal reaction (desmoplasia) [87,88]. In this desmoplastic response, PDACs exhibit a marked proliferation of stromal fibroblasts and deposition of extracellular matrix components [89,90]. The role of this process in pancreas carcinogenesis remains in question, since it is not well established whether the response is part of the tumorigenic program or whether it represents a form of host defense against the tumor. Recent evidence suggests that the stroma may contribute to tumor growth through paracrine signaling, ECM remodeling, and angiogenesis [91,92,93,94]. Our previous investigations concluded that SMAD4 deficiency altered the histological phenotype of KrasG12D-initiated neoplasms. While KrasG12D alone initiated PanIN development that progressed slowly to PDAC, the combination of mutant KrasG12D and SMAD4 deficiency in the mouse pancreas resulted in the development of tumors resembling human intraductal papillary mucinous neoplasia (IPMN) [31,95]. SMAD4 deficiency also accelerated PDAC development in KrasG12D combined with INK4A/ARF heterozygous-loss mice and affected the histological type of PDAC with a more highly differentiated type, rather than a poor differentiate tumor [13].

### 5.2. Ptf1a-Cre, KrasG12D/+, TβR2^L/L^ Mice

TGFβ is thought to promote PDAC desmoplasia (stromal proliferation), as well as contribute to the proliferation and invasion of the tumor cells in an autocrine manner; notably, the blockade of TGFβ signaling attenuates tumorigenicity of some xenografts [88,96]. In contrast, the tumor suppressor role of TGFβ signaling is underscored by presence of inactivating TGFβ receptor mutations in many different cancers, including colorectal and pancreatic cancer [77,97,98,99] (Figure 3). Significantly, the role of TGFβ in blocking cancer development also involves control of epithelial cell/tissue microenvironment interactions, as demonstrated by the development of epithelial cancers in mice with T-cell specific Smad4 deletion and fibroblast-specific TGFβRII deletion. Pancreas-selective Tgfbr2 knockout alone in mice revealed no discernable phenotype in mice. However, pancreas-specific Tgfbr2 knockout combined with Kras^G12D^ expression (Ptf1a-cre Kras^G12D^ Tgfbr2^Lox/Lox^ mice) developed well-differentiated PDAC at 8–10 weeks of age with 100% penetrance. Heterozygous deletion of Tgfbr2 with Kras^G12D^ expression also developed well differentiated PDAC with higher frequency of liver, duodenum, and lung metastases as compared to Ptf1a-cre; LSL-Kras^G12D/+^; Tgfbr2^Lox/Lox^ mice [100]. Further intensive investigation of this model may be required to provide a better understanding of specific subtype of PDAC, in which TGF-β signaling and activated Ras signaling cooperate to promote disease progression.

### 5.3. Pdx1-Cre, KrasG12D/+, ACVR1B^L/L^ Mice

TGFβ signal is an important biological tumor suppressor program that is based on the prevalent genomic deletion of the TGF-β superfamily gene in pancreatic cancer [101]. Recently, it has been identified that aberrations in the TGFβ superfamily pathway, whether through the BMPs, Activin, or TGFβ receptor of the pathway, can result in tumorigenesis and promote the tumor progression. Activin, one of the TGFβ superfamily members, plays many important roles in PDAC carcinogenesis. Results from cancer genome-sequencing studies revealed that the activin A receptor Type 1B (ACVR1B) gene is mutated around 2% of PDAC samples, which may imply that ACVR1B could be a tumor suppressor gene in PDAC [102,103] (Figure 3). Meanwhile, Activin signaling alterations are also implicated in cancer development. For instance, a recent study indicated that human PDAC samples markedly over-expressed the activin/inhibin beta A subunit, whereas the beta B subunit was only moderately increased in comparison to normal pancreatic samples using in situ hybridization analysis. The activin signaling causes growth inhibition and apoptosis principally through SMAD4-dependent pathways in numerous cancers. In a conventional knockout mouse model of Acvr1b, the recent study demonstrated embryonic lethality due to developmental impairment of the epiblast and extraembryonic ectoderm, causing to abnormal gastrulation [104]. Furthermore, a conditional knockout of ACVR1B in the pancreas increased the proliferation of pancreatic epithelial cells, promoted to the formation of ADM (acinar to ductal metaplasia), and induced pancreatic inflammation [105]. Disruption of Acvr1b cooperating with Kras accelerated the development of cysts that resembled intraductal papillary mucinous neoplasm, but did not alter the growth of pancreatic intraepithelial neoplasias. Lastly, they also found that loss of the p16 (Inka 4a) gene expression might be required for progression of IPMNs to pancreatic ductal adenocarcinomas in Acvr1b^Lox/Lox^, Kras^G12D^, Pdx1-Cre mice [105].

### 5.4. Pdx1-Cr, KrasG12D/+, KLF10^L/L^ Mice

KLF10, the zinc finger transcription factor, is a member of the Krüppel-like family of transcription factors. KLF10 can be induced by estrogen, TGF-βs, BMP, NGF, and EGF [106,107,108,109,110,111]. Recently, a study showed that TGF-β1 regulated KLF10 transcription to inhibit epithelial cells’ proliferation and induce cell apoptosis after TGF-β1 stimulation, demonstrating that KLF10 is a critical component for transducing TGF-β1 signaling [112,113,114] (Figure 3). One study found that KLF10 induces apoptosis in the TGF-β signaling pathway of resistant cancer cells and concurrently increases chemosensitivity for treatment with gemcitabine [115,116]. Furthermore, others found Klf10 silencing correlates with radiation resistance in pancreatic cancer by up-regulator UVRAG (UV radiation resistance-associated gene) [117]. Recently, we demonstrated the potential tumor-suppressing function of KLF10 in the progression of pancreatic cancer, via employing two well-characterized Pdx-1 Cre, LSL-Kras^G12D^ and Pdx-1Cre, LSL-Kras^G12D^, p53^Lox/Lox^ PDAC models, combined with KLF10 loss to dissect the molecular mechanism underlying KLF10 loss on pancreatic tumor development and progression [118]. In our study, we showed that loss of KLF10 cooperates with Kras^G12D^ leading to an invasive and widely metastatic phenotype of PDAC. Our studies further revealed that loss of KLF10 increased distant metastases and cancer stemness through activation of SDF-1/CXCR4 and AP-1 pathways in PDAC [118]. 

### 5.5. Pdx1-Cre, KrasG12D/+, TGIF1^L/L^ Mice

The TG-interacting factor 1 (TGIF1), a nuclear transcriptional corepressor of the TGFβ/Smad signaling, has been associated in the pathogenesis of numerous types of cancer (Figure 3). A recent study demonstrated that TGIF1, a component of ubiquitin ligase, mediates the degradation of Smad2 in the TGF-β signaling and the recruiting corepressor complex containing histone deacetylases (HDAC) to modify chromatin structure [119,120,121]. Moreover, other studies have exposed that TGIF1 also opposes early stages of TGF-b signaling, probably by limiting access of phosphorylation Smad2 by TβRI [122,123]. In general, TGIF1 induces the expression of I-Smad, increasing the Smad7 expression, which interferes with the TGFβ receptor by blocking the interactions between the R-Smad and the receptor complex [119]. The other report showed that TGIF1 suppressed of R-Smad gene expression by inhibitory cofactors Sno, Ski, and TGIF1 [120,124]. More specifically, the conditional deletion mouse model of TGIF1 in pancreas did not show an observable impact on pancreatic development or physiology [125]. Notably, loss of TGIF1 combined with KrasG12D promoted shorter latency PDAC and a greater propensity for distant metastases. We also founded that TGIF1 might function as an epigenetic regulator and response for aberrant EMT gene expression during PDAC progression [125]. Intriguingly, our results also revealed a novel role of TGIF1 in suppressing the expression of PD-L1, an immune checkpoint molecule. Taken together, we demonstrated that key effectors of TGIF1 loss in murine PDAC biology and determined that TGIF1 loss led to more aggressive immune suppression, EMT-high, and elevated stemness gene signature phenotypes of PDAC [125].

## 6. Loss of APC or Activation of Wnt/β-Catenin in PDAC

Pdx1-Cre, KrasG12D/+, APC^L/+^ mice. Apc modulates the canonical Wnt signaling pathway through mediating β−catenin degradation, thus inactivation of APC creates a permissive condition, whereby free unphosphorylated β-catenin is significantly more stable, and translocates into the nucleus to active Wnt signaling [99,126,127,128]. Many studies reported that Wnt signaling is activated at high levels during early pancreatic development. Meanwhile, activation of Wnt signaling predominately in acinar cells results in an increase in pancreas mass [129,130]. Additionally, ectopic activation of Wnt signaling at early stages of pancreas organogenesis may regulate the increases of Hedgehog activity, while Hedgehog signaling is known to active embryonic activity in many endodermal organs [131,132]. Further, upregulation of Wnt signaling could be induced via specific mutations in the APC, β-catenin, or Axin genes, which are thought to play some crucial roles in the development of human gastrointestinal tumors [133,134]. Mutations in either APC or β-catenin are commonly found in other gastrointestinal cancer, including PDAC [135,136]. Recent studies reported that aberrant cytoplasmic and nuclear expression of β-catenin, both indicative of canonical Wnt signaling activity, are present in a substantial group of PDAC and PanIN samples [137]. Furthermore, Lewis and his colleagues demonstrated that the Ptf1a (p48)-Cre LSL-KrasG12D elastase-tva compound mice injected with fibroblasts, producing RCAS viruses encoding the Wnt1 gene, commonly developed large uni or multilocular mucin producing cysts (MCNs), with high expression of Wnt in the mouse pancreas [138]. These data imply that Wnt signaling plays vital role in PDAC, despite the absence of clear signature mutations in APC or β-catenin so far. Nevertheless, activation of the Wnt signaling pathway in pancreatic cancer has remained controversial, thus functional studies addressing a potential contribution of APC mutation and Wnt/β-catenin signaling to PDAC development and progression are anticipated to provide essential insights in this area. From our previous study, attempting to study the deletion of APC in the pancreas, we observed that APC haploinsufficiency corresponded with Kras^G12D^ mutation and P53 loss in mice, leading to rapidly increased development of metastatic PDAC when compared with Pdx1-cre, KrasG^12D^, P53^Lox/+^ mice That evidence indicated a critical tumor suppressor role of APC in pancreatic cancer progression [139].

## 7. Alterations of DNA Damage Response and Chromatin Remodeling Regulators in PDAC

**Figure 4:**
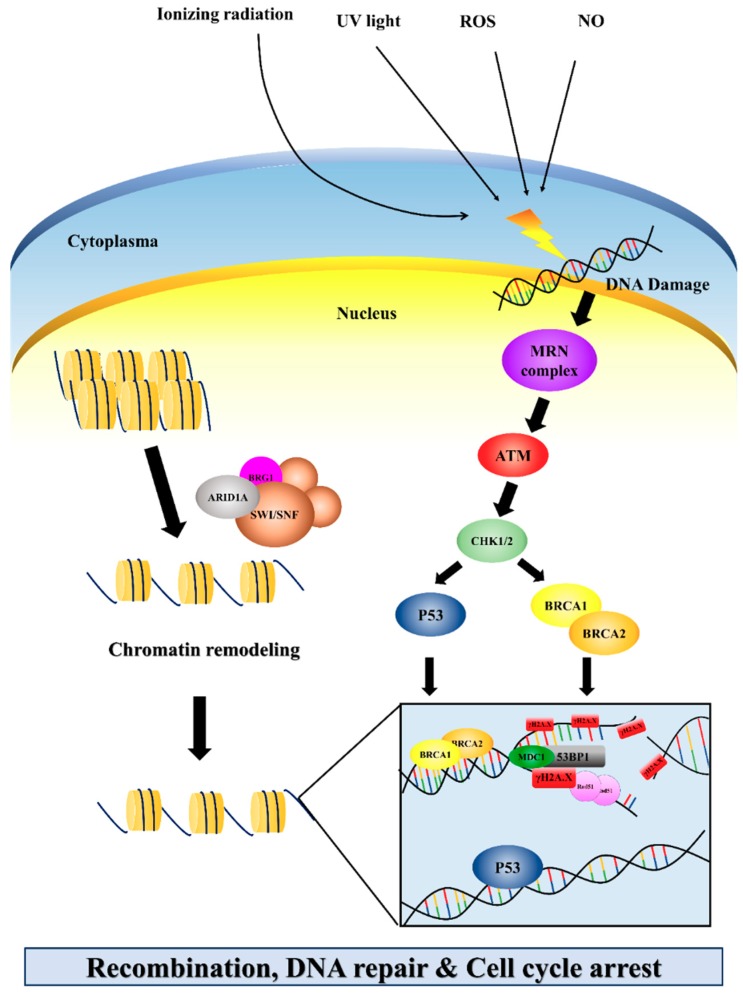
Schematic depiction and overview of the chromatin remodeling and DNA repair pathways of ARDA1a, BRG1, ATM, BRCA1, and P53 after DNA damage. Examples of the molecules known to act on regulatory pathways are listed. Note that some critical components of the pathways have been omitted for clarity.

DNA damage sometimes results in multiple broken chromosomes, which can be caused by many factors, such as ionizing radiation (IR) and ultra violet light (UV), and by various metabolic compounds within cells (including reactive oxygen species (ROS) and nitric oxide (NO)) [140,141]. DNA damage involves single- and double-strand breaks (SSB and DSB respectively), the formation of pyrimidine dimmers (PD), and oxidized nucleotides [142,143]. When cells active DNA repair processes to repair such damage, chromatin constitutes a physical barrier to the repairing machinery to reach the DNA. To accomplish this, histones can be modified by chromatin remodeling complexes, such as SWI/SNF (BAF) chromatin remodeling complexes, to make chromatin more accessible to conduct the base- and nucleotide-excision, homologous recombination, and non-homologous end joining DNA repair pathways [144,145]. Arid1a (adenine-thymine rich interactive domain 1) protein is known to respond by directing the SWI/SNF complex to target promoter regions, and it regulates the transcription of certain genes by altering the chromatin structure [146] (Figure 4). SWI/SNF is a multi-subunit complex utilizing the energy of ATP hydrolysis to reposition nucleosomes in order to orchestrate transcription factors to bind to DNA and regulate the expression of genes. Human SWI/SNF complexes contain either of two alternative catalytic (ATPase) subunits, SMARCA4 (BRG1) or SMARCA2 (BRM), as well as 8–10 other subunits [147,148]. Meanwhile, genes encoding subunits of SWI/SNF chromatin remodeling complexes are collectively altered in approximately 20% of human cancers [149,150]. Arid1a participates in the regulatory loops modulating p53-dependent, and E2F-dependent cell survival, and damage/stress pathways [151]. Arid1a has also been shown to directly interact with p53, a tumor suppressor gene that controls cell growth arrest or apoptosis after DNA damage, to modulate p53 regulatory pathway in cancer cells [152]. In addition, ARID1A is one of the most frequently deleted genes in a variety of tumor types, and knockdown of ARID1A leads to a failure of cell cycle arrest [152]. Notably, there are many of the proteins involved in the activation of the DNA strand break repair and damage signaling, including ATM, Mdc1, 53BP1, Rad51, and the MRN-Rad50-Nbs1 complex, colocalized with γH2Ax in DNA repair foci (Figure 4). DNA double-strand breaks (DSBs) activate the ATM kinase and induce a cascade of DNA damage signals by the phosphorylation of hundreds of proteins involved in cell cycle checkpoint activation, DNA repair, and apoptosis, including p53, Chk2, γH2Ax, BRCA1, and Nbs1 [153,154,155] (Figure 4). Deletion of ATM function in mammal cells causes impairment in DNA repair functions and cell cycle checkpoint control. Not surprisingly, humans and mice with loss of ATM function are prone to carcinogenesis [156,157,158]. Breast cancer type 1 susceptibility protein (BRCA1) is a nuclear protein that functions as a tumor suppressor with distinct patterns of cell-cycle-regulated expression and is hyperphosphorylated to sense DNA-damage, in order to direct DNA repair [159,160]. Meanwhile, germline mutations in BRCA1 and BRCA2 confer increased risks to hereditary breast and ovarian cancer syndrome [160,161]. A number of studies have shown that pedigrees with germline mutations in ATM, BRCA1, and BRCA2 also have an increased lifetime risk of pancreatic cancer in familial pancreatic cancer (FPC) kindreds [103,162,163,164,165,166]. Subsequently, evidence from the following conditionally deficient mice revealed the potential roles of these chromatin remodeling and DNA repair genes in maintaining genomic stability and preventing the risk of developing pancreatic neoplasms.

### 7.1. Pdx1-Cre, KrasG12D/+, Brca2 Tr/Δ11 Mice

The BRCA1 and BRCA2, tumor suppressor genes, play a critical role in the repair of DNA double-strand breaks and homologous recombination (HR) pathway, and is involved in maintenance of structural and numeric chromosomal stability as well [159,160]. Notably, germline mutations in BRCA1 and BRCA2 were originally identified in patients with hereditary breast ovarian cancer syndrome. [160,161]. However, Brca1 and Brca2 are also involved among the most common genetic lesions in familial pancreatic cancer patients. One study found that mice with conditional germline homozygosity for Brca2 truncating mutations led to developing PanINs at five months of age, and 15% of the mutant mice progressed to pancreatic cancer at 15 months of age without Kras activation [30]. Furthermore, it has been reported that germline heterozygosity for BRCA2 and K-ras^G12D^ cooperate to promote PDAC progression and systemic metastasis in mice [167]. In this report, tumor growth and survival time were observed and compared in mice with Pdx1-Cre, LSL-K-ras^G12D^ and a germline heterozygosity for Brca2 ^Tr/Δ11^ background, and Pdx1-Cre, LSL-K-ras^G12D^ mutant mice, and they found that the median survival for Pdx1-Cre, LSL-K-rasG12D, Brca2 Tr/Δ11 mice is significantly shorter by approximately 5 months. In contrast, in another study to demonstrate the function of Brca2 in the progression of pancreatic cancer, the mouse models that combined pancreas specific KrasG12D activation and TP53 deletion with BRCA2 inactivation were generated. Unexpectedly, their results indicated that the mutant model of TP53 deletion and Brca2 inactivation promoted pancreatic cancer development, but the mouse model of KrasG12D combined with BRCA2 inactivation caused chromosomal instability and cell apoptosis, and resulted in the inhibition of tumors growth [167].

### 7.2. Ptf1a-Cre, KrasG12D/+, ATM^L/L^ Mice

ATM (ataxia telangiectasia-mutated) is the crucial DNA damage sensor of the response to DNA double strand breaks, which plays the tumor suppressor role in the DNA damage checkpoint pathway as p53 after DNA double-strand breaks [168,169]. Loss of ATM was identified by the increased mitotic defects, recurrent genomic rearrangements, and deregulated DNA integrity checkpoints. Germline mutations in ATM may cause increased risks in developing familial pancreatic cancer, and somatic mutations in ATM also have been reported in resected numerous sporadic human pancreatic cancer [165]. To study the loss of ATM associated with the genome integrity during PDAC progression, a recent report demonstrated the potential effects of Atm deletion in the pancreatic cancer mouse model. This report revealed that conditional deletion of ATM in a mouse model combined with Kras^G12D^ of pancreatic cancer induces more proliferative precursor lesions coupled with pancreatic fibrosis in the mice [170]. Furthermore, ATM-null PDAC mice displayed altered BMP4 signaling, and promoted the epithelial-to-mesenchymal transition (EMT), coupled with shortened survival. On the other hand, other studies indicated that ATM deficiency increases the proportion of chromosomal alterations in pancreatic cancer. They also found that ATM deficiency renders murine pancreatic cancer highly sensitive to radiation treatment. These findings suggested that the ATM signaling pathway poses a major barrier to pancreatic carcinogenesis via maintaining the genomic stability [171].

### 7.3. Ptf1a-Cre, KrasG12D/+, ARID1a^L/L^ Mice

The chromatin remodeler Switch/Sucrose nonfermentable (SWI/SNF)-complex functions as a tumor suppressor in many human malignant cancers [172,173,174]. Results of several recent studies revealed that mutations, translocations, and deletions of various subunits of the SWI/SNF complex occur in approximately 20% of human cancers, thus representing one of the most common altered molecular mechanisms in human cancers [150,175]. Importantly, the AT-rich interactive domain 1A (ARID1A) gene which encodes the BAF250a protein, is the most frequently mutated of the SWI/SNF subunit, and has been reported to be mutated in nearly 20%–23% of pancreatic cancer cases [9,176]. Arid1a protein is thought to influence transcription factor associations with the SWI/SNF complexes, and was recently postulated to act as a tumor suppressor and a driver gene in PDAC [146]. Notably, Arid1a has been identified with a DNA-binding ARID domain, which can specifically bind to a DNA sequence that is AT-rich and associated with other transcriptional factors [177]; and it also confers specificity to the SWI/SNF complex and recruits the chromatin remodeling complex to its targets by binding to proteins and to DNA. Meanwhile, Arid1a is implicated in ATM/ATR initiated DNA repair and ARID1A loss leads to deficient in DNA repair and vulnerability to DNA damage. One study done in GEM models revealed that conditional knockout of ARID1A induces inflammation, PanINs, and mucinous cysts [178,179]. Furthermore, ARID1A loss cooperates with Kras to accelerate the development of cysts that resemble intraductal papillary mucinous neoplasm. Their results further revealed that ARID1A loss induced pancreatic neoplasia associated with the increased Myc activity and enhanced protein translation [179].

### 7.4. Ptf1a-Cre, KrasG12D/+, BRG1^L/L^ Mice

The SWI/SNF complex functions as a crucial regulator of gene expression by recruiting to specific DNA regions and interacts with various transcription factors. The SWI/SNF complex was able to shift the position of histones of the chromatin in an ATP-dependent manner, and to give transcription factors access to bind the DNA, thereby regulating transcription [148,180]. It is important to note that the SWI/SNF complex has been shown to involve a wide variety of cellular processes, such as development, cell growth, differentiation, drug resistance, and DNA repair. In humans, the SWI/SNF chromatin remodeling complex is composed of two distinct ATPase subunits; one is SMARCA4 (BRG1) and the other is SMARCA2(BRM) [181]. Brg1/SMARCA4 encodes a catalytic core subunit of the SWI/SNF complex, which is an essential transcription factor in the regulation of chromosome structures. In general, mutations in the SWI/SNF complex have now been found in about 20% of all human cancers [182]. Notably, one-third of pancreatic cancers harbor deletions or mutations in several subunits of the SWI/SNF complex [183]. Recently, numerous studies explored novel functions of BRG1 in cancers, and showed that Brg1 functions as a tumor suppressor. Furthermore, decreased BRG1 expression is reported to be correlated with the progression, occurrence, and poor prognosis of breast, gastric, ovarian, lung, and pancreatic cancers [184]. In pancreatic duct cells, a recent report demonstrated that Brg1 inhibits the dedifferentiation that precedes neoplastic transformation in adult pancreatic ductal cells [185]. Meanwhile, results from genetically mouse models revealed that conditional deletion of BRG-1 in the pancreas from the mouse model of mutant KrasG12D induced PDAC resulted in the formation of MCNs or IPMNs, and some progress to PDAC [185,186]. In contrast, restoration of Brg1 expression leads to induce the mesenchymal phenotype in mouse and human BRG-1 null PDAC cells. Thus, Brg1 might have bipolar contextual roles, both preventing and enhancing pancreatic tumorigenesis in a stage-dependent manner [185,186].

### 7.5. Pdx1-Cre, KrasG12D/+, P53^L/L^ Mice

The P53 tumor suppressor gene is mutated in greater than 50% of PDAC samples [29]. The p53 protein mediates growth arrest or apoptosis upon insult to the integrity of chromosomal DNA, dependent on the levels of DNA damage and the cellular stress [187,188]. For instance, research studies demonstrated that the ATM protein could specifically phosphorylate p53 at multiple serine residues, and those modifications are dependent on γ radiation, radiomimetic drug induced DNA damage, or UV irradiation. Meanwhile, many studies have shown that P53 mutations appeared in advanced PanINs, consistent with a role in malignant PDAC progression. In contrast to many other cancer types, there does not appear to be a reciprocal relationship in the loss of INK4a/ARF and P53, pointing to non-overlapping functions for ARF and P53 in pancreatic cancer suppression [189]. P53 loss is thought to contribute to the rampant genetic instability that characterizes this malignancy; i.e., profound aneuploidy and complex cytogenetic rearrangements, as well as intratumoral heterogeneity consistent with ongoing genomic instability [190]. Moreover, correlations have been reported in human PDAC between p53 mutations and enhanced VEGF expression, increased angiogenesis, and higher MVD, with all correlations to poor prognosis in human PDAC [191,192]. Thus, we suspected that this capability of p53 to suppress angiogenesis may be one reason that p53 is frequently inactivated in PDAC already deficient in function of the P16/INK4A and P14/ARF tumor suppressors. In the genetically mouse-model settings, expression of an activated Kras (KrasG12D) knock-in allele in the epithelium induces PanIN lesions that can gradually progress to PDAC (average latency > 1 year) [7]. Homozygous deletion of the P53 tumor suppressor locus alone does not induce pancreatic neoplasia; however, concurrent KrasG12D expression and p53 nullizygosity drives the development of highly aggressive PDAC within 8–12 weeks [14,15]. These results demonstrated that Kras activation acts as an initiating event in PanIN-to-PDAC progression, while p53 functions to constrain the malignant progression of PanINs into well differentiated metastatic PDAC [24].

## 8. Conclusions and Future Perspectives

PDAC is the deadliest of common human adult malignancies [1]. The vast majority of patients present with unresectable disease, and have virtually no hope for cure or even long-term survival. This advanced clinical presentation has also resulted in extremely limited tissue resources for biological investigations of these tumors. Despite significant advances in the past two decades in the chemotherapeutic management of human malignancies, there has been only very slight impact on the extremely poor median survival of patients with PDAC. Recent studies of engineered mouse models of de novo pancreatic cancer, characterized in unprecedented detail, the aberrant lesional microenvironment and its malignant regulation by genetic mutations during multistage progression. These models define the genetic basis of human PDAC, establish and characterize new mouse models of PDAC development, and define the biological basis of the initiation, development, and progression of mouse and human pancreatic neoplasms from early PanINs through to invasive and metastatic PDACs (Table 1). Thus, these genetically engineered mouse models (GEMMs) are powerful research platforms for oncology research, and are uniquely positioned to characterize the morphological phenotypes of early neoplasms, relate these findings to epithelial biology, and rapidly advance our understanding of mouse models and human malignant disease, and/or metastatic progression. The understanding produced by these PDAC models should ultimately lead towards the development of novel therapeutic strategies for this deadly disease.

## Figures and Tables

**Table 1:** List of mouse models studying driver genes in pancreatic cancer.

Models	Tumor Histology	Tumor Time Course	Gene Alteration Condition	Cre Type	References
Kras^G12D^; P53^R172H/+^	PDAC	PanIN (4–6 weeks) PDAC (4.5m)	Tp53R172H	Pdx1-cre	[14]
Kras^G12D^; SMAD4 ^Loxp/Loxp^	IPMN	PanIN (4 weeks) IPMN (8 weeks)	Exon 8–9	Ptf1a-cre	[31]
Kras^G12D^; LKB1 ^Loxp/+^	PDAC	PanIN (6 weeks), PDAC (20 weeks)	Exon 3–6	Pdx1-cre	[66]
Kras^G12D^; ATM ^Loxp/Loxp^	PDAC	PanIN (10 weeks)	Exon 57–58	Ptf1a-cre	[170,171]
Kras^G12D^; BRG1 ^Loxp/Loxp^	IPMN	IPMN (9 weeks)	Exon 18	Ptf1a-cre	[185,186]
Kras^G12D^; INK4A/ARF^Loxp/Loxp^	PDAC	PDAC (7.9 weeks)	Exon 2–3	Pdx1-cre	[7]
Kras^G12D^; KLF10^Loxp/Loxp^	PDAC	PDAC (20–24 weeks)	Exon 1–2	Pdx1-cre	[118]
Kras^G12D^; TGIF1^Loxp/Loxp^	PDAC	PDAC (18–20 weeks)	Exon 2–3	Pdx1-cre	[125]
Kras^G12D^; BRCA^Δ11/Δ11^	No tumor	N/A	Exon 11	Pdx1-cre	[193]
Kras^G12D^; ARID1A^Loxp/Loxp^	IPMN	IPMN (8 weeks)	Exon 8	Ptf1a-cre	[178]
Kras^G12D^; EGFR^KO^	No PanINs	N/A	Exon 1	Ptf1a-cre	[46]
Kras^G12D^; PTEN^Loxp/+^	PDAC	ADM and PanIN (4–6 weeks), PDAC (17 weeks)	Exon 5	Pdx1-cre	[63,64]
Kras^G12D^; TGFBR2^Loxp/Loxp^	PDAC	PDAC (6–7 weeks)	Exon 4	Ptf1a-cre	[100]
Kras^G12D^; ACVR1B^Loxp/Loxp^	IPMN	ADM (2m), IPMN (3m)	Exon 2–3	Pdx1-cre	[105]
Kras^G12D^; P53^Loxp/+^; APC^Loxp/+^	PDAC	PDAC (6–7 weeks),	Exon 15	Pdx1-cre	[139]
